# Splenic-preserving surgery in hydatid spleen: a single institutional experience

**DOI:** 10.25122/jml-2021-0221

**Published:** 2022-01

**Authors:** Samer Makki Mohamed Al-Hakkak, Ashraf Sami Muhammad, Saad Ab-Razq Mijbas

**Affiliations:** 1. Department of Surgery, Jabir Ibn Hayyan Medical University, Najaf, Iraq; 2. Department of Surgery, College of Medicine, Kufa University, Kufa, Iraq

**Keywords:** spleen, hydatid cyst, echinococcosis, preserving spleen, splenectomy

## Abstract

Though hydatid disease affects many organs in the human body, splenic hydatid accounts for approximately 0.8-4% of all human echinococcosis cases. Up to recently, splenectomy was the preferred surgery for hydatid spleen. Since 1980, conservative options to treat such a disease have become more and more prevalent. Our study aimed to assess our experience in open splenic preservative surgery for splenic hydatid in a single institutional center. Our retrospective research included ten patients with splenic hydatid operated between August 2013 and January 2018 at our medical center. The spleen was affected alone in seven cases, the liver and spleen were affected in three cases, and one of the patients had intra-peritoneal cyst disease. The diagnosis was confirmed primarily by ultrasonography. In some instances, computed tomography and magnetic resonance imaging were required. A chest x-ray was performed to rule out pulmonary hydatid in all patients. Open surgery procedure, field isolation, cystic fluid aspiration, and injection of 1% cetrimide solution, respiration, endocystectomy, suture of cystic edges to the intracystic tube drain were performed. All surgeries had albendazole before and after the operation 15 mg/kg/day. There were no significant intra or postoperative complications, and no further surgery was required. Patients remained hospitalized for 3-5 days. No recurrence after 1-3 follow-up years. However, three patients failed to follow up within two years. Our experience with splenic hydatids prompts us to use splenic conservation surgery whenever possible instead of splenectomy.

## Introduction

The most common form of hydatid disease in humans is caused by Echinococcosis granulosa (EG); it is a localized cystic disease found most commonly in the liver and lungs. Other forms of hydatid disease are uncommonly caused by E. Multilocularis, E. Vogleri, and E. Oligarthrus [1, 2]. Hydatid disease is a zoonotic infection causing significant public health problems in many areas worldwide, particularly among the population that practices sheep husbandry [3, 4]. The prevalence of the disease has been declared high in Middle Eastern countries, particularly in Iraq, because of sheep and dogs living in close contact with humans, particularly among rural people and Bedouins [5]. The embryo of E. granulosus escapes from the eggs, penetrates the intestinal mucosa, enters the portal, and is transported to various organs, mostly the liver 75% (the first filter interrupted organ), the lungs 15 % (the second filter interrupted organ), the remaining organs such as spleen, kidney, and others. There are several speculations regarding the possible routes to reach the spleen; the arterial route seems to be the most logical and could account for its infrequent appearance since to follow this path, the parasites would have to cross both hepatic and pulmonary filters. The other pathway is transcoelomic spread to other organs, mainly the superficial hydatid cyst rupture of the liver [6]. Some researchers believe that the embryo can migrate from the portal vein to the splenic vein, affecting the spleen when there is an increase in intra-abdominal pressure [7]. Embryos may also make their way into the lacteals and pass directly into systemic circulation through cisterna chyli [1]. Isolated hydatid of the spleen is rare, even in endemic regions, constituting 0.8–4% of all cases of human hydatid while it constitutes 5.8 % of all cases of abdominal hydatid [8, 9]. It represents the third most commonly involved organ in the body after the liver and lung [10]. The condition is characterized by its latency; the cyst gradually increases over several years before reaching a size that could be clinically detected [11]. However, the onset of complication brought the disease into an overt state during any period of cyst development [12] which includes cystic rupture in the peritoneal cavity, which produces an omental or visceral cyst. In the past, the spleen was considered an unnecessary organ because it was believed to be not essential to life; increased understanding of the adverse effects of splenectomy has led to conservatism in managing the splenic disease. Therefore, surgeons try to preserve the spleen and maintain the functions that will be further described. The spleen’s immunologic function is a major site for specific immunoglobulin M (IgM) production. The filtering function in which reticulum macrophages capture cellular and non-cellular material from the blood, including bacteria, particularly pneumococcus. “Culling” is another essential function of removing old platelets and old red cells.

The spleen plays a major role in removing red cell nuclei and malaria parasites without destroying the cell. Iron reutilization is another splenic function. The spleen can act as a pooling organ since up to 30–40% of the blood platelets are sequestrated within the spleen, leading to post-splenectomy thrombocytosis [13]. The postoperative complication of splenectomy is due to surgical procedures such as damage to the pancreatic tail, gastric or non-technical fistula such as post-splenectomy septicemia or post-splenectomy opportunistic infection (OPSI) [13]. Bacterial infections with septicemia are rapidly progressing to circulatory collapse and death following splenectomy [14], especially in young children. Abrupt onset, the rapid development of shock, and frequent accompaniment of meningitis are characteristic of overwhelming post-splenectomy sepsis. It occurs more frequently in younger patients, but it may occur in older patient-years after splenectomy [15, 16]. Streptococcus pneumoniae is the most common cause of septicemia in children and splenectomized adults, representing 60% of the cases. Neisseria meningitidis and Haemophilus influenzae type b represent another 25%. The remainder is caused by Escherichia coli, streptococci, staphylococcal auras, Klebsiella, and salmonella. Splenectomized patients face an increased risk of post-splenectomy opportunistic infection (OPSI), so Pneumovax, anti-toxin pneumococcal, should be given two weeks before surgery. It is important to advise the patient of the dangers of OPSI and prescribe antibiotics for all infections. Splenectomized patients living in malaria-endemic areas should receive antimalarial prophylaxis [13].

## Material and Methods

Between August 2013 and January 2018, ten patients with splenic hydatid had splenic-preserving surgery in our center. Eight of them were retrospectively studied, and two were followed prospectively. All patients were subjected to clinical evaluation, taking a complete history, physical examination, and complete investigation into account. Diagnosis of splenic hydatid was primarily confirmed by ultrasonography. Computed tomography (CT), magnetic resonance imaging (MRI) were required in some instances, and chest radiography allowed all patients to exclude pulmonary hydatid. A non-complicated hydatid cyst diagnosis depended on clinical suspicion when the patients presented with upper abdominal pain, mainly in the left upper quadrant or epigastrium, the most common splenomegaly symptoms.

Inclusion criteria were age group between 5–40 years, intact splenic hydatid cyst, spleen hydatid cyst or spleen and liver hydatid cyst. Exclusion criteria were hydatid disease (multiple organs affected hydatid), under 5 years and above 40 years, complicated splenic hydatid, a fully calcified cyst. Ultrasound and computed tomography, cyst classification based on Gharbi classification (Table 1 and 2) were performed [14]. This was initially developed for liver hydatid cysts based on ultrasound results. It later expanded to include CT results and other organs such as the spleen.

**Table 1.** Gharbi’s classification for hydatid cyst.

**Type I**	Univesicular cyst
**Type II**	Univesicular cyst with membrane detachment
**Type III**	Multivesicular cyst
**Type IV**	Pseudotumoral cyst
**Type V**	Fully calcified cyst

**Table 2.** Type of splenic hydatid cyst according to Gharbi’s classification.

**Type of splenic hydatid cyst according to Gharbi’s classification**	**Number (%)**
Type I	4 (40%)
Type II	3 (30%)
Type III	3 (30%)
Type IV	0
Total 10	10 (100%)

Preoperative preparation was based on the patient’s general condition and the ability for anesthesia. Laparotomy was made under general anesthesia, supine position, abdomen was opened either through the midline or left under the costal incision, followed by identification of splenic hydatid cyst. Before surgery, exploration of the peritoneal cavity for undetected hepatic or peritoneal hydatid was performed. Isolation of the surgical field by packages was equipped with hypertonic solution (20–30% hypertonic saline). The cystic fluid was aspirated. A suitable amount of approximately 5 ml of solution (1% cetrimide) was injected, followed by a minimum of 5 minutes wait, then re-spired. The spleen was not required for mobilization, removal of the final cyst, and washing the cavity with normal saline, occupying the cystic cavity of the intracystic tube drain. There was careful preoperative and post-op monitoring of the patient in case of anaphylaxis or any other complication. The patient was monitored until discharge from the hospital 3–5 days, by ultrasound in the first postoperative month and then six months after operation, then yearly until three years. Three patients failed to follow up within two years. All surgical operations were carried by the same surgical team. All operations were carried out under the cover of albendazole 15mg/kg/day preoperative during one week and continued postoperatively for 3 months in 3 cycles, each one 28 days, with rest one week between each cycle and continuation afterward.

## Results

Six of these men and four women were between the ages of 5 and 40 years (mean 23.6 years). The hydatid spleen alone was present in 7 cases, while splenic and hepatic hydatid was present in 3 cases, one of which had a peritoneal hydatid disease. The hospital stay ranged from 3 to 5 days. There were no significant intraoperative complications or challenges, significant postoperative morbidity, and no mortality. One patient developed fever due to lung atelectasis and was appropriately treated. One patient developed a wound infection postoperatively. There was no recurrence after 1–3 years of monitoring, although three patients were not followed after two years. Ultrasound monitoring imaging showed a decrease in the size of the cyst bed with the disappearance of the component, the appearance of pseudotumor, and the cystic wall becoming irregular and thickened. Follow-up of patients over the 1–3 year period revealed complete cavity obliteration and successful spleen retention, as shown in Tables 3 and 4.

**Table 3.** Demographic and clinical characteristics of the study patients.

**Surgical features**	**Spleen-preserving surgery, n (%)**
**Number of patients**	10
**Male**	6 (60%)
**Female**	4 (40%)
**No symptoms**	3 (30%)
**Left upper abdominal pain**	7 (70%)
**Serological tests positive**	7 (70%)
**Unilocular cyst**	7 (70%)
**Multilocular cyst**	3 (30%)
**Spleen and liver affection**	2 (20%)
**Spleen alone**	7 (70%)
**Spleen, liver, and intraperitoneal**	1 (10%)
**Cyst in upper pole of spleen**	2 (10%)
**Cyst in middle part of spleen**	2 (10%)
**Cyst in lower pole of spleen**	6 (60%)
**Cyst diameter (3–5) cm**	8 (80%)
**Cyst diameter >5 cm**	2 (20%)

**Table 4.** Description of the postoperative complications.

**Surgical features**	**Spleen-preserving surgery, n**
**Pleural effusion**	0
**High platelet syndrome**	0
**Adhesive partial small-bowel obstruction**	0
**Pneumonia**	1 (10)
**Residual cavity effusion**	0
**Recurrence (1–3 years)**	0
**Wound infection**	1 (10)
**Total**	2 (10)

## Discussion

The spleen is one of the uncommon organs affected by hydatid disease. Up to recently, splenectomy was the recommended treatment for spleen hydatid. However, increasing knowledge of the untoward effects of splenectomy [13], mainly the risk of post-splenectomy sepsis (immunological function) and operative risk, made it important to save the splenic parenchyma and its function as much as possible [17] and led to conservatism in the management of the splenic disease [18, 19].

For this reason, the surgeons now try to spare the spleen and maintain its functions. The spleen’s most common benign cystic lesion is hydatid, mainly caused by EG [19]. Although it is uncommon, the treatment is mandatory because of the severity and frequency of complications. Nowadays, attempted preservation of the spleen and maintaining function is mandatory when treating a benign illness [19–21]. Cystic inoculation and drainage of splenic hydatid should always be attempted [22], splenectomy should only be performed when local conditions require it, or technical mistakes occur. Despite the encouraging results in our patients, the number of patients included in the study was limited. Therefore, an adaptation of this option as safe and effective should be investigated in multicenter studies, assessing the results of this surgical option. Hydatid spleen cyst is a rare condition, and spleen preservation should always be attempted, especially in children. To date, surgery continues to be the cornerstone of the treatment of echinococcosis, including splenic echinococcosis. Splenectomy reports showed benefits in terms of reduced recurrence and fewer complications [23–26].

## Conclusion

Surgery remains the first line of treatment of hydatid cysts. Our experience demonstrated the feasibility of removing the hydatid cyst of the spleen while preserving the splenic functions. Thus the potential catastrophe of post-splenectomy complication can be avoided. Splenectomy should only be conducted when required by local conditions or when technical errors occur.

## Acknowledgments

### Conflict of interest

The authors declare that there is no conflict of interest.

### Ethical approval

The study obtained ethics approval from the ethical committee of the Faculty of Medicine, Jabir Ibn Hayyan Medical University (320 JMU-6^th^ March 2020)

### Consent to participate

Written informed consent was taken from all patients.

### Authorship

SMMA-K is the corresponding author and was in charge of collecting data, manuscript concept, writing, data analysis, and submitted manuscript revision. ASM was in charge of data collection. SARM was in charge of data collection and data analysis.
